# Preparation and Evaluation of Green Composites from Microcrystalline Cellulose and a Soybean-Oil Derivative

**DOI:** 10.3390/polym9100541

**Published:** 2017-10-23

**Authors:** Wendi Liu, Ming-en Fei, Yang Ban, Anming Jia, Renhui Qiu

**Affiliations:** College of Transportation and Civil Engineering, Fujian Agriculture and Forestry University, Fuzhou 350108, China; wdliu9054@163.com (W.L.); 3161317001@fafu.edu.cn (M.-e.F.); 18065043547@163.com (Y.B.); anmingnir@163.com (A.J.)

**Keywords:** microcrystalline cellulose, acrylated epoxidized soybean oil, mechanical properties, water absorption, interfacial adhesion

## Abstract

The present work aimed at developing fully green composites from renewable materials, i.e., acrylated epoxidized soybean oil (AESO) and microcrystalline cellulose (MCC) by a solution casting method. The reinforcing effect of MCC on AESO resins was optimized by adjusting MCC loading from 20 to 40 wt % in terms of physical, mechanical, and thermal properties as well as water absorption of the resulting MCC/AESO composites. The interaction between MCC and AESO was characterized by Fourier transform infrared (FTIR) analysis, which revealed possible hydrogen bonds between the –OH groups of MCC along with the polar components of AESO including C=O, –OH, and epoxy groups. This was further evidenced by a benign interfacial adhesion between MCC and AESO resins as revealed by scanning electron microscope (SEM) analysis. The incorporation of MCC into AESO resins significantly increased the density, hardness, flexural strength, and flexural modulus of the MCC/AESO composites, indicative of a significant reinforcing effect of MCC on AESO resins. The composite with 30 wt % MCC obtained the highest physical and mechanical properties due to the good dispersion and interfacial interaction between MCC and AESO matrix; the density, hardness, flexural strength, and flexural modulus of the composite were 15.7%, 25.0%, 57.2%, and 129.7% higher than those of pure AESO resin, respectively. However, the water resistance at room temperature and 100 °C of the composites were dramatically decreased due to the inherent hydrophilicity of MCC.

## 1. Introduction

**Scheme 1:**
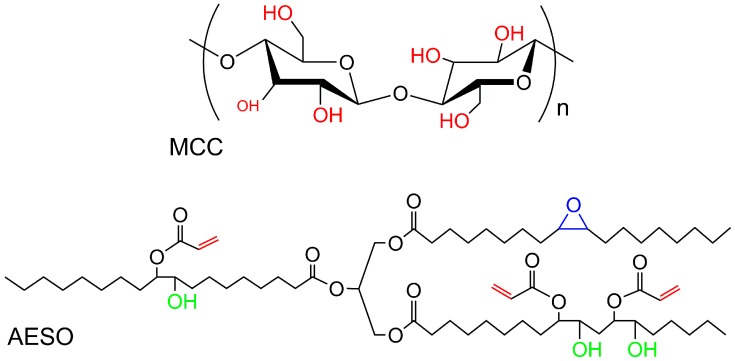
Chemical structure of microcrystalline cellulose (MCC) and acrylated epoxidized soybean oil (AESO).

High-performance materials derived from renewable resources have generated great interest due to the ever-increasing environmental and sustainable issues. Cellulose is one of the most abundant and renewable biopolymers on earth. Cellulose is the main structural component of the primary cell wall of many green plants [1]. Cellulose is a linear chain composed of repeating d-glucopyranose connected together by β-1,4-glucosidic linkages (Scheme 1); the adjacent anhydroglucose molecule chains are coupled with each other by a large amount of hydrogen bonds and Van der Waals forces to generate a stable and heterogeneous supramolecular structure [2]. Microcrystalline cellulose (MCC) is a key form of the hydrolyzed cellulose consisting of extensive cellulose microcrystals together with amorphous regions. MCC is much stronger and stiffer than amorphous, or even, original cellulose [3]. MCC is usually isolated from various cellulosic resources by mechanical [4], biological [5], and chemical treatments [6]. MCC is stable, nontoxic, and chemically inactive. Other potential advantages of MCC include renewability, biodegradability, low density, and high specific strength and surface area. Therefore, MCC has been widely applied in various fields including food packages, pharmaceutical formulations, and composite industries [5,7,8].

MCC has been utilized as a reinforcing agent in thermoplastic and thermosetting resins. The main challenge for the fabrication of MCC-reinforced polymer composites is the aggregation of MCC within polymer matrices, which stems from the inherent incompatibility between the hydrophilic MCC and the hydrophobic polymer matrices. This bottleneck was normally addressed through addition of coupling agents into the matrices or surface chemical treatments of MCC to reduce its hydrophilicity. Polypropylene (PP) composites reinforced with MCC were developed by using maleic anhydride (MA) [9] and MA-grafted-PP (MAPP) [3,10] as coupling agents, respectively. The MCC was acetylated with ketene and vinyl acetate, respectively, for the preparation of MCC-reinforced polyethylene composites [11,12]. The grafting of MCC with l-lactic acid oligomers was performed for improving the interfacial adhesion between MCC and polylactic acid [13]. The grafting copolymerization of MCC with (meth)acrylic monomers is an effective method in improving the dispersion of MCC in natural rubber [2,14]. The MCC-reinforced unsaturated polyester (UPE) composites were prepared via in-situ polymerization, indicating that the addition of MCC into UPE systems significantly increased the dynamic mechanical properties of the UPE resins [15]. Pure MCC was also used as reactive reinforcing agent for the preparation of MCC-reinforced polyurethane (PU) composites because the hydroxyl groups of MCC would react with the isocyanate groups of PU resin systems [16,17]. In summary, numerous routes for MCC modification and preparation of MCC-reinforced composites with different polymer matrices have been widely studied; however, most of these works focused on the use of petroleum-based polymers as matrices in the MCC composites.

The development of biobased polymer matrices from renewable resources, such as vegetable oils, carbohydrates, and proteins, provides an effective way to reduce the consumption of oil resources in cellulose-reinforced composites [18,19]. Vegetable oils are thought as ideal raw materials to prepare sustainable polymers because they are abundant, readily available, and inexpensive. As one of the main soybean oil derivatives, acrylated epoxidized soybean oil (AESO), contains three C=C bonds which is able to copolymerize with styrene for the formulation of AESO-based thermosets (Scheme 1). Styrene is required for forming a mixture with low viscosity for wetting reinforcing fibers because AESO is highly viscous at room temperature. Some styrene-AESO composites have been prepared from various reinforcing materials including carbon nanotube [20], glass fiber [21], layered silicate [22,23], and natural fibers [24,25,26]. However, styrene is potentially carcinogenic [27]. Further, the large use of nonpolar styrene in AESO matrices would give the resins with a high hydrophobicity and thus incompatible with the hydrophilic cellulose fibers.

In this study, MCC was introduced into AESO system without reactive diluents for the preparation of cellulose-reinforced AESO composites by a solution casting method. AESO contains many polar groups, such as esters, hydroxyl groups, and epoxy rings, which might form hydrogen bonding with the hydroxyl groups of MCC and hence an improved interfacial adhesion of the resulting MCC/AESO composites. The reinforcing effect of MCC with different loadings from 20 to 40 wt % on AESO matrix together with the interaction between MCC and AESO were investigated in terms of the physical properties, flexural properties, water absorption, thermal stability, crystalline structure, and interfacial adhesion of the MCC/AESO composites.

## 2. Materials and Methods

### 2.1. Materials

Microcrystalline cellulose (MCC, average particle size: 50 µm) was obtained from Thermo Fisher Scientific (Waltham, MA, USA). Acrylated epoxidized soybean oil (AESO, average molecular weight: 1200 g/mol; viscosity (25 °C): 18,000–32,000 mPa·s; acid value: ≤10 mg KOH/g; inhibitor: 3500–4500 ppm monomethyl ether hydroquinone), *tert*-butyl peroxybenzoate (TBPB, 98%), and acetone (≥99.9%) were purchased from Sigma-Aldrich (St. Louis, MO, USA). All the chemicals were used as received.

### 2.2. Preparation of MCC/AESO Composites

MCC powder was dispersed in acetone in a 250 mL round bottom flask that was immersed into an ultrasonic water bath (ultrasound power: 80 W) and then stirred magnetically with a rate of 500 rpm at room temperature for 5 min. Then, AESO was dissolved into the MCC-dispersed solution at different weight ratios of MCC to AESO, i.e., 20/80, 30/70, and 40/60, which were denoted as 20MCC, 30MCC, and 40MCC, respectively. The resulting mixture was mechanically stirred at 80 °C for 10 min to uniformly disperse MCC with AESO resins and to remove acetone from the solution and then cooled to room temperature. Afterward, 2 wt % TBPB (based on AESO) was added into the obtained MCC-AESO mixture that was stirred at room temperature for 2 min and then transferred to silicon molds (80 × 10 × 3 mm^3^) to prepare the cured composite samples. The curing reaction was performed at 120 °C for 2 h, followed by 160 °C for another 4 h in an oven. Pure AESO sample was also prepared for comparison as the same procedure. After being cooled to room temperature, the samples were removed from the mold for testing.

### 2.3. Characterization

The chemical compositions and possible interactions between MCC and AESO in the composites were revealed with attenuated total refraction Fourier transform infrared (ATR-FTIR) analysis. The MCC, cured AESO and composite samples were ground into powders for FTIR tests on a PerkinElmer Spectrum One Spectrometer (PerkinElmer, Waltham, MA, USA) equipped with 3× bounce diamond crystal and an incident angle of 45°. Spectra were collected under the following conditions: 4000–650 cm^−1^ range; 4 cm^−1^ resolution; 16 scans.

X-ray diffraction (XRD) analysis was conducted on a Bruker D8 Advance diffractometer (Bruker, Billerica, MA, USA) fitted with a Lynxeye XE high-resolution energy dispersive 1-D detector. The X-ray unit was operated at 40 kV and 40 mA using a Ni-filtered Cu-Kα radiation of 0.1542 nm. Angular scanning was conducted from 5° to 40° at 0.2°/s and the data were collected using DIFFRAC.SUITE software (Bruker, Billerica, MA, USA).

The densities of pure AESO and MCC/AESO composites were determined according to the relationship of mass/volume. The mass of the samples was measured in a balance with a precision of 0.001 g. The volume was obtained by pouring the samples in water in a 50 mL graduate cylinder. Five replicates were measured for each group of sample. The hardness of samples was measured using an ISH-DSA Digital Shore Durometer A (INSIZE, Loganville, GA, USA). The samples were placed on a flat surface, and a cycle time of 3 s was used for the measurement. Ten replicates were taken per sample.

Flexural properties of the resin and composites were evaluated in comply with ASTM D 790-10. Ten rectangular specimens (80 × 10 mm^2^) were tested by using a CMT6104 microcomputer controlled electronic universal testing machine (MTS Systems, Eden Prairie, MN, USA) at a crosshead rate of 5 mm/min.

Water absorption of the resin and composites were measured by immersing rectangle samples (80 × 10 mm^2^) in distilled water at room temperature and 100 °C, respectively. Before testing, the samples were conditioned in an oven at 50 °C for 3 h and then cooled down to room temperature. Then, the samples were weighed and immersed in distilled water at room temperature for 24 h. Afterward, the samples were removed from water, wiped with tissue paper, and weighed to measure the weight gain. Similarly, the samples were immersed in a boiling water bath for 2 h to determine water absorption. Then, the samples were removed from the boiling water and cooled in distilled water for 15 min at room temperature before measuring the weight gain. The water uptake rate of the composites was calculated as the weight gain divided by the dry weight of the specimen. Five replicates were measured for each composite.

Thermogravimetric (TG) analysis of the resin and composites were performed on a DTG-60 TG instrument (Shimazu, Kyoto, Japan) at a scan rate of 10 °C/min from room temperature to 600 °C in N_2_ atmosphere (flow rate: 50 mL/min). Samples (4–6 mg) were placed in aluminum crucibles by using an empty aluminum crucible as reference.

The distribution of MCC and its interfacial adhesion with AESO matrix were examined with a Zeiss Supra 35 VP field emission scanning electron microscopy (FEG-SEM, ZEISS Microscopy, Jena, Germany) at an operation voltage of 10 kV. Samples were coated with elemental gold film (8–10 nm) before testing.

## 3. Results and Discussion

### 3.1. ATR-FTIR Analysis

**Figure 1:**
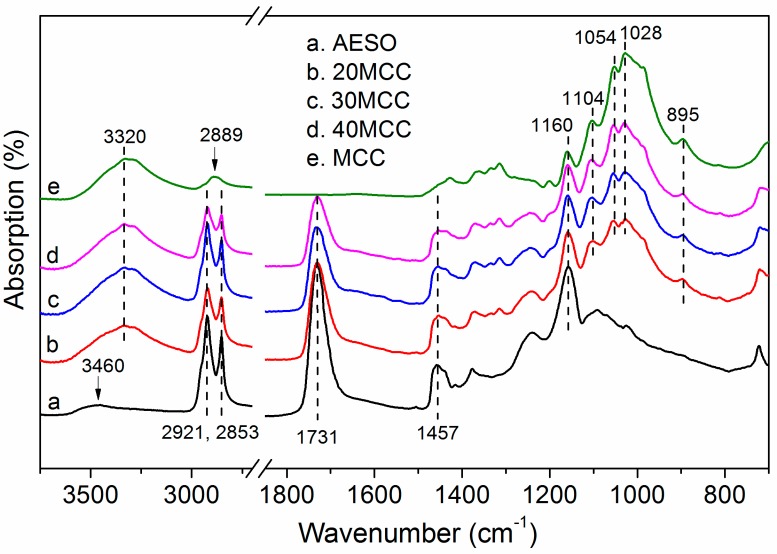
Fourier transform infrared (FTIR) spectra of pure MCC, AESO, and MCC/AESO composites with different MCC loadings.

The FTIR spectra of pure AESO, MCC, and MCC/AESO composites are shown in Figure 1. The spectrum of AESO displays characteristic peaks at 3460, 1731, and 1241 cm^−1^, representing the stretching vibrations of –OH groups, C=O, and C–O groups of ester, respectively (Figure 1a) [28,29], which are the main polar components of AESO molecules. The bands originated from C–O–C stretching vibration of ester are situated at 1160 and 1092 cm^−1^ [29]. The peaks at 2921 and 2853, and 1457 and 1378 cm^−1^ are due to the asymmetric stretching vibrations and deformation of C–H in the –CH_2_– and –CH_3_ bonds, respectively, which are from the inherent aliphatic sequences of AESO [30,31]. By contrast, the –OH and H–bonding stretching of cellulose is apparent at around 3320 cm^−1^ in the spectrum of MCC (Figure 1e). The peak at 2889 cm^−1^ is attributed to the stretching vibration of –CH_2_– bonds [31]. The bands at 1160, 1054, and 895 cm^−1^ are associated with the C–O–C asymmetrical stretching at β-glucosidic linkages, C–O–C pyranose ring skeletal vibration, and β-glucosidic linkages of amorphous cellulose, respectively [32,33,34]. The –COH stretching vibrations of primary (C6) and secondary (C2 and C3) alcohols from glucose units are located at 1028 and 1104 cm^−1^, respectively.

Different compositions in the MCC/AESO composites could be found from the FITR spectra of the composites containing different MCC concentrations (Figure 1b–d), which presents the characteristic peaks resulted from the combination of those of MCC and AESO. The intensity of typical peaks of AESO at 2921, 2853, 1731, and 1457 cm^−1^ are dramatically decreased after the incorporation of MCC; however, those of MCC at 1104, 1054, 1028, and 895 cm^−1^ are slightly increased with the addition of MCC from 20 to 40 wt %. However, there are not any new peaks appearing in the spectra of the composites, except for the main characteristic signals of MCC and AESO, which suggests that the added MCC has no covalent bonds with AESO.

**Figure 2:**
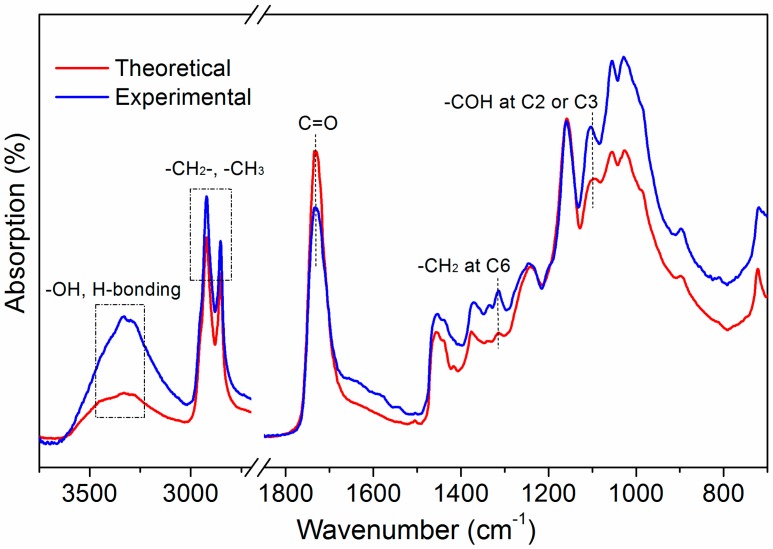
Comparison of experimental FTIR spectra of composite 30MCC with its theoretical curve calculated from pure MCC and AESO based on Lambert-Beer’s law.

The possible interactions between MCC and AESO in the composites could be revealed by comparing the experimental FTIR spectrum of 30MCC to its theoretical curve calculated from the spectra of pure AESO and MCC based on Lambert-Beer’s law as the following equation [35]:

*A*_30MCC_ = 0.3*A*_MCC_ + 0.7*A*_AESO_ (1)

where *A*_30MCC_*, A*_MCC_ and *A*_AESO_ are the absorbances (%) in the spectra of 30MCC, MCC and AESO, respectively; and the coefficients are the weight ratios of MCC and AESO in the composites. As presented in Figure 2, it is observed that the intensities of –CH_2_– and –CH_3_ bonds from AESO in the experimental spectrum are much stronger than those in the theoretical one, which is probably due to the fact that, as a matrix, AESO prefers to coat on the surface of MCC during the preparation of MCC/AESO composites. However, the change in C=O groups of AESO shows a reverse trend with that in aliphatic chains. Further, the bands at 3460–3320 cm^−1^ indicating the –OH and H–bonding stretching are significantly stronger in the experimental spectrum than the theoretical one. These are related to the possible hydrogen bonds between the –OH groups of MCC with the polar components of AESO including C=O, –OH, and epoxy groups, which results in lower C=O stretching and higher –OH and H–bonding stretching in the experimental spectrum. Additionally, compared with those of the theoretical spectrum, the peaks at 1314 and 1104 cm^−1^, which are corresponding to the –CH_2_ rocking at C6 and the –COH stretching at C2 or C3, respectively [36], are stronger in the experimental spectrum. This might be contributed by the ultrasound treatment during composite preparation which is effective in breaking the hydrogen bonds between glucose units and thus exposing more –OH groups on the MCC surface.

### 3.2. XRD Analysis

**Figure 3:**
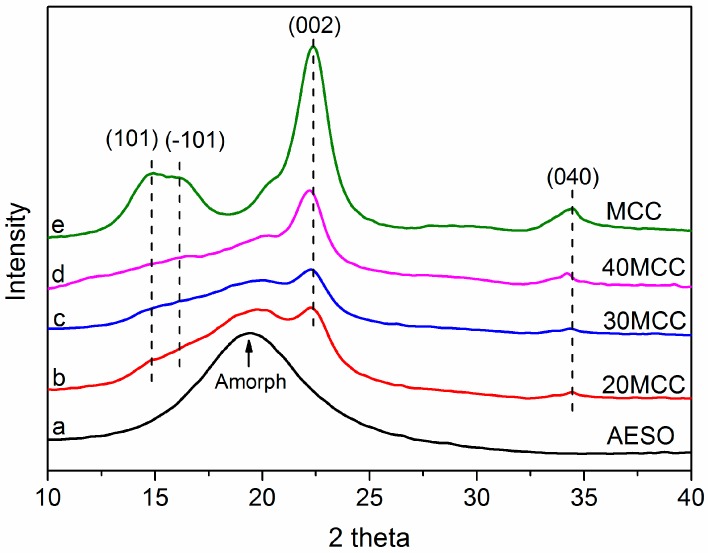
XRD patterns of pure MCC, AESO and MCC/AESO composites with different MCC loadings.

The typical XRD patterns of pure MCC, AESO, and MCC/AESO are given in Figure 3. The spectrum of AESO only shows a strong peak at around 19.4°, representing the amorphous materials of AESO [37]. However, MCC contains peaks at around 14.9°, 16.2°, 22.3°, and 34.5°, which are typical for native cellulose I structure corresponding to the (101), (10-1), (002), and (040) lattice planes, respectively [38,39]. After the incorporation of MCC into AESO, the XRD patterns of the composites show the characteristic peaks of the two components. The intensity of the diffraction peaks (002) and (040) resulted from MCC significantly increases with the increasing in MCC loading, indicating an increased crystalline region in the composites.

### 3.3. Physical Properties

The densities of neat AESO and MCC/AESO composites are presented in Table 1. The use of MCC for reinforcing AESO significantly increases the density of the resulting composites with respect to the neat AESO (*P* < 0.05), which is expected because the density of MCC (1.46 g/cm^3^) is much higher than that of AESO [40]. The density of the composites increases with the increase of MCC usage from 20 to 30 wt %; however, further increase in MCC loading to 40 wt % results in the reduction in density of the composites. This might be related to the improved void content of the composites with high MCC content [41]. The agglomeration of MCC is easy to occur in a composite with excess MCC content as revealed in SEM analysis (Section 3.7), which would prevent the uniform penetration of resin matrix through reinforcement, hence leading to the formation of poor interfacial adhesion between resin and MCC and increasing the porosity within the composites [42].

As given in Table 1, compared with that of neat AESO, the hardness values of composites 20MCC, 30MCC and 40MCC are increased by 27.0%, 25.0%, and 24.6%, respectively, which is contributed by the MCC with high stiffness. However, the increase of MCC amount in AESO matrix has not significant impact on the hardness of the composites (*P* > 0.05), which might be due to the poor penetration of AESO resin onto MCC when high content of MCC was applied.

### 3.4. Flexural Properties

**Figure 4:**
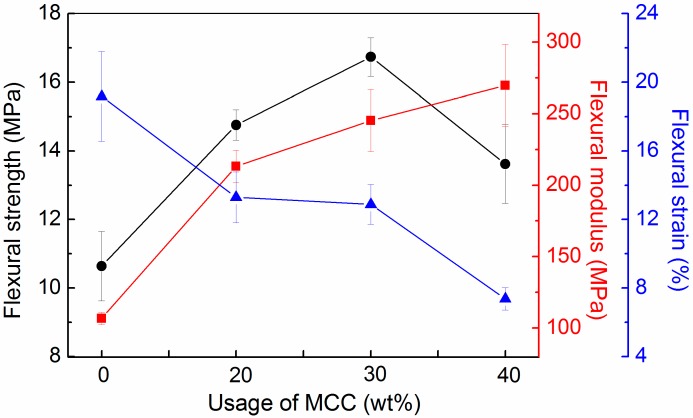
Effect of MCC loading on the flexural properties of MCC/AESO composites. (● Flexural strength; ■ Flexural modulus; ▲ Flexural strain).

It is apparent that the reinforcing of MCC in AESO results in significant improvements in the flexural strength and flexural modulus of the resulting MCC/AESO composites (*P* < 0.001) (Figure 4). The flexural strengths of the composites with 20 and 30 wt % MCC greatly increase by 38.7% and 57.2%, respectively, compared with that of neat AESO. However, further increase in MCC loading to 40 wt % leads to a dramatic reduction in the flexural strength of the composites (*P* < 0.01). The addition of MCC as a reinforcing agent in AESO might contribute to improving the mechanical strength of the composites because MCC would effectively absorb majority of the loading when the materials are loaded. The decrease in flexural strength at high MCC content is probably caused by the agglomeration of cellulose and insufficient propagation of resin through fillers, which would form voids and micro-cracks within the composites and hence reducing the loading distribution efficiency and stress transfer in the composites [43]. On the other hand, compared with that of neat AESO, the flexural moduli of the composites greatly increase by 106.5%, 129.7%, and 152.6% with the incorporation of 20, 30, and 40 wt % MCC, respectively. This is expected because MCC has a straight rigid chain structure and is much stiffer than flexible AESO matrix, which induces stiffness to the resulting composites [44]. However, after the incorporation of MCC into AESO, the flexural strain of the composites is decreased, accompanied by the increase of flexural strength and modulus (*P* < 0.001). Due to the insufficient crosslinking density, the pure AESO resin contains a large amount of flexible AESO molecular chains, which are beneficial for dispersing instantaneous loading and effectively prevent stress concentration, thus showing superior flexural strain [45]. However, this effect is eliminated after the use of MCC in the resin because MCC provides high rigidity and modulus for the composites, hence giving rise to the brittleness of the materials. It is reported that the MCC-reinforced composites from soybean oil-based polyurethane and epoxy resins exhibited tensile strengths of 6~22 MPa and tensile moduli of 200~600 MPa, but their flexural properties were not available in these works [16,17,43]. Compared to the reported soybean oil–based composites, the obtained MCC/AESO composite in this study does not use any petroleum-based hardeners, including anhydrides and isocyanates, and hence is a relatively green composite from renewable resources.

### 3.5. Water Absorption

**Figure 5:**
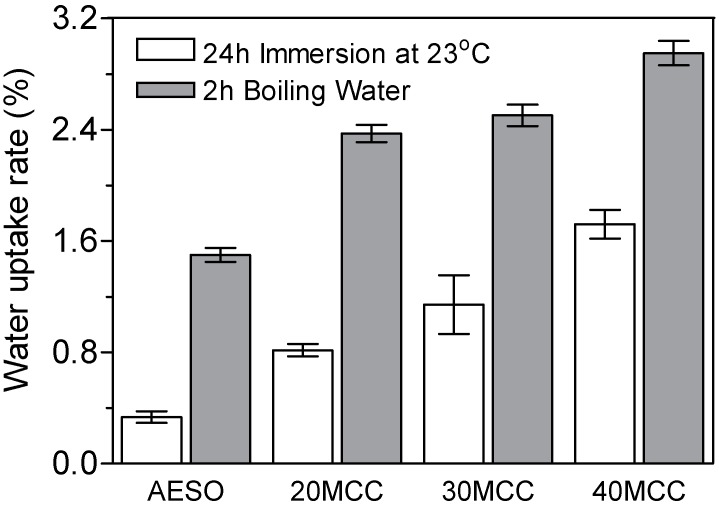
Effect of MCC loading on the water absorption of MCC/AESO composites.

The water uptake rates of pure AESO and MCC/AESO composites at room temperature (RT) for 24 h and an elevated temperature (boiling water) for 2 h are shown in Figure 5. For all the samples, the water uptake rates at 100 °C are much higher than those at room temperature (*P* < 0.001). The higher water absorption in boiling water is attributed to the higher diffusivity of water molecules into the composites at high temperature, which results in more cracks induced by water at an accelerated rate [46]. Especially, for the composites immersed in a high temperature environment, the development of microcracks on the surface and interface region of the composites is considerably accelerated by the more active water molecules [47]. The water absorption rates of neat AESO at RT and 100 °C are 0.34% and 1.50%, respectively, which is closely connected with the polar components of AESO molecules such as –OH, epoxy and ester groups. It is obvious that the water absorption percentages of all composites with different MCC loadings at both environments are much higher than those of the neat AESO (*P* < 0.001). This is due to the fact that MCC contains a large number of hydroxyl groups on the surface and thus is highly hydrophilic. As confirmed by FTIR analysis, there are possible hydrogen bonds between the –OH groups of MCC with the polar groups of AESO in the composites. However, this bonding is weak and easily destroyed by the attack of water molecules, and as a result, the –OH groups of both MCC and AESO are exposed and form hydrogen bonds with the water molecules. Furthermore, the water absorption percentages of MCC composites at two temperatures are significantly affected by MCC concentration. The water uptake percentages of the composites at RT significantly increase with the increase of MCC usage from 20 to 40 wt % (*P* < 0.001); however, the water absorption percentages of composites 20MCC and 30MCC at 100 °C are statistically comparable (*P* > 0.05), and both of them are significantly lower than that of composite 40MCC (*P* < 0.001). This can be explained by the following three reasons: (1) For the composites with low amount of MCC usage (20 and 30 wt %), the MCC can be completely covered by AESO resin, which effectively prevents the direct contact of water molecules with MCC; (2) for the composite with excess MCC (40 wt %), the aggregation of MCC and its insufficient wetting by resin would create MCC-MCC interface inside the composites, which provides a good route for water penetration between MCCs; (3) the increased MCC usage also contributes to more free –OH groups in the composites for absorbing water.

### 3.6. Thermal Stability 

**Figure 6:**
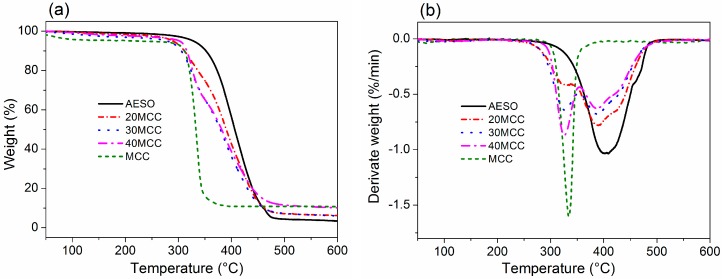
TG and DTG curves of pure MCC, AESO, and MCC/AESO composites with different MCC loadings.

The thermal degradation of pure AESO, MCC, and MCC/AESO composites are presented in Figure 6. The maximum weight loss temperature (*T*_max_) are tabulated in Table 2. Pure AESO shows a broad degradation peak on the DTG curve, which is closely related to the main chemical functionalities of AESO including aliphatic chains and ester groups. By contrast, the TG and DTG curves of MCC indicate a fast degradation of the β-1,4-glucosidic linkages of cellulose chains. The *T*_max_ of pure AESO is much higher than that of MCC, indicating a higher thermal resistance of AESO with regard to MCC. The thermal degradation of polymers refers to the cleavage of organic functional groups such as C–C, C–O, and C=O bonds. The aliphatic chains of AESO has higher thermal resistance than the C–O bonds of cellulose, which contributes to a reduced thermal stability of the composites after the incorporation of MCC into AESO resins. A weight loss peak at low temperature (*T*_max1_) appeared on the DTG curves of the MCC/AESO composites when compared with that of pure AESO, which is majorly attributed to the thermal degradation of MCC. The addition of MCC also reduces the *T*_max2_ of the composites which is mainly resulted from the decomposition of AESO. Both *T*_max1_ and *T*_max2_ of the composites decrease with the increase of MCC loading, which indicates reduced thermal stability of the composites.

### 3.7. SEM Analysis

**Figure 7:**
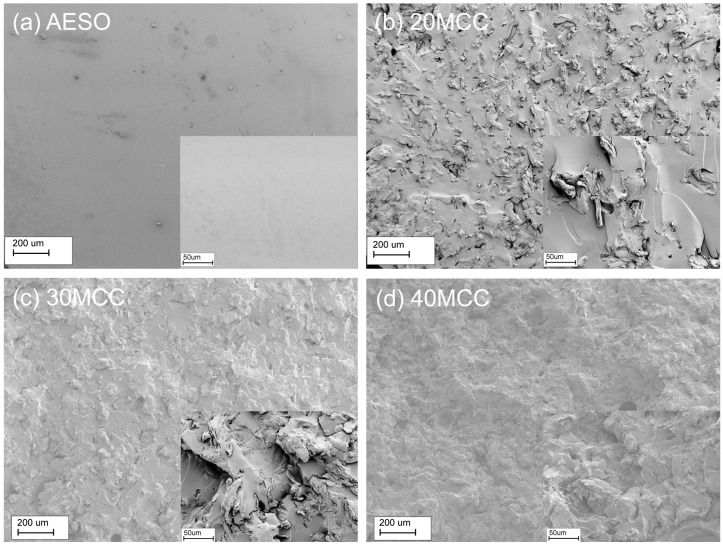
Scanning electron microscope (SEM) images of pure AESO and MCC/AESO composites with different MCC loadings.

The SEM images from the flexural-fractured surface of the AESO composites reinforced with different usages of MCC are presented in Figure 7. The surface of pure AESO is substantially smoother than those of the composites. The surface of composite 20MCC shows that MCC is uniformly dispersed in the composites; an ambiguous MCC-matrix interface is observed on the surface, which indicates a superior interfacial adhesion between MCC and AESO due to the hydrogen bonding. The integrity of AESO matrix is further destroyed when increasing MCC loading to 30 and 40 wt %. The incorporation of substantial MCC into AESO matrix results in the aggregation of MCC and the appearance of obvious holes resulted from MCC pull-out on the surface.

## 4. Conclusions

Green composites with superior properties were successfully prepared from renewable materials, i.e., cellulose and soybean oil derivative, by a simple solution casting method. The obtained MCC/AESO composites with 20 to 40 wt % MCC loadings showed higher density, hardness, flexural strength, and flexural modulus than pure AESO resin due to the reinforcing effect of MCC. An optimum MCC concentration of 30 wt % was obtained for the composites, and the composites had 15.7%, 25.0%, 57.2%, and 129.7% higher density, hardness, flexural strength, and flexural modulus, respectively, than pure AESO resin. However, the thermal stability and water resistance of the composites decreased after the incorporation of MCC because MCC has a low thermal decomposition temperature and high hydrophilicity. The FTIR spectra of the composites indicated characteristic peaks for MCC and AESO and revealed possible hydrogen bonding between MCC and AESO. The SEM images of the composites with 20 wt % MCC showed a good interfacial interaction between MCC and AESO due to the hydrogen bonding.

## Figures and Tables

**Table 1:** Physical properties of pure AESO and MCC/AESO composites with different MCC loadings.

Composites	Density (g/cm^3^)	Hardness (HA)
AESO	1.02 ± 0.09	53.70 ± 3.21
20MCC	1.09 ± 0.03	68.20 ± 2.31
30MCC	1.18 ± 0.02	67.10 ± 1.78
40MCC	1.13 ± 0.01	66.90 ± 7.50

**Table 2:** Maximum weight loss temperatures of pure MCC, AESO, and MCC/AESO composites with different MCC loadings.

References	Maximum Weight Loss Temperatures (°C)	
	*T*_max1_	*T*_max2_
AESO	-	405.8
20MCC	326.8	389.5
30MCC	325.3	387.7
40MCC	325.1	386.2
MCC	334.0	-
